# Parameter-dependent cell-type specific effects of transcranial focused ultrasound stimulation in an awake head-fixed rodent model

**DOI:** 10.1088/1741-2552/adbb1f

**Published:** 2025-03-19

**Authors:** Sandhya Ramachandran, Huan Gao, Eric Yttri, Kai Yu, Bin He

**Affiliations:** 1 Department of Biomedical Engineering, Carnegie Mellon University, Pittsburgh, PA, United States of America; 2 Neuroscience Institute, Carnegie Mellon University, Pittsburgh, PA, United States of America; 3 Department of Biological Sciences, Carnegie Mellon University, Pittsburgh, PA, United States of America

**Keywords:** neuromodulation, transcranial focused ultrasound, awake head-fixed rodent model, cell-type specificity, pulse repetition frequency, duty cycle

## Abstract

*Objective.* Transcranial focused ultrasound (tFUS) is a promising neuromodulation technique able to target shallow and deep brain structures with high precision. Previous studies have demonstrated that tFUS stimulation responses are cell-type specific, and specifically tFUS can elicit time-locked neural activity in regular spiking units (RSUs) that is sensitive to increases in pulse repetition frequency (PRF), while time-locked responses are not seen in fast spiking units (FSUs). These findings suggest a unique capability of tFUS to alter circuit network dynamics with cell-type specificity; however, these results could be biased by the use of anesthesia, which significantly modulates neural activities. *Approach.* In this study, we developed an awake head-fixed rat model specifically designed for simultaneous tFUS stimulation using a customized 128-element ultrasound array transducer, and recording of spiking data. Using this novel animal model, we examined a series of PRFs and burst duty cycles (DCs) to determine their effects on neuronal subpopulations without anesthesia. *Main results.* We observed cell type specific responses to varying PRF and DC in the awake setting as well as the anesthetized setting, with time locked responses observed in RSU and delayed responses in FSU. Anesthesia broadly was found to dampen responses to tFUS, and affected the latency of delayed responses. Preferred parameters for inducing time-locked responses appear to be 1500 Hz PRF and 60% DC. *Significance.* We conclude that despite some differences in response, isoflurane anesthesia is not a major confound in studying the cell-type specificity of ultrasound neuromodulation, but may affect studies of circuit dynamics and FSU. Our developed awake model will allow for future investigations without this confound.

## 1. Introduction

Neuromodulation techniques aim to treat neurological disorders non-pharmacologically by precisely stimulating neural circuits. Many techniques aiming to do this have been studied [1–7], but have limitations due to invasiveness, limited spatial resolution, and limited ability for deep brain penetration. Low-intensity transcranial focused ultrasound (tFUS) is a noninvasive neuromodulation technology using focused ultrasound pressure waves [8–13], with high spatial resolution [9, 14] and the ability to target deep brain structures [15, 16]. Recent studies additionally suggest the ability to affect different cell-types through parameter selection [17, 18], making tFUS a promising neuromodulation modality.

Previous studies of these features are limited by their use of anesthetized rodent models. The anesthetized brain has reduced neural activity and responsivity [19], and isoflurane in particular is known to alter potassium and calcium channels, as well the GABA receptor complex in inhibitory neurons [20–22]. Several theories [23, 24] concerning the mechanism of tFUS involve modulation of ion channel properties, which these anesthesia effects would likely interact with [25, 26]. Anesthesia also causes varying responsivity to stimuli over time, potentially affecting the variance of tFUS responses through a recording session [27–29].

These effects may confound our previous finding of cell type specific parameter sensitivity [30–34]. Recent work has demonstrated that parameters such as the pulse repetition frequency (PRF) and the duty cycle (DC) play an important role in tuning the tFUS effect [18, 31, 35], and vary in effect between cell subtypes [18, 36]. A large range of these parameters have been tested without a clear consensus due to the wide range of stimulation paradigms and targets. Essentially the entire range of DCs have been studied although those under 50% are more common due to heating concerns. PRFs from 15 Hz to 1 MHz have also been tested, with the majority testing between 300–1000 Hz [8]. Our group observed that in the somatosensory cortex (S1), only putative excitatory neurons have a time-locked response to tFUS that is sensitive to the change of PRF. Selective stimulation between excitatory and inhibitory neurons would open possibilities for a variety of therapeutic treatments, but this effect is confounded by anesthesia. These cell types are differently affected by anesthesia [22], and studies in S1 may generally be affected by anesthetic dampening of thalamo-cortical connectivity [19, 37, 38]. Due to these confounds, it is critical to test these findings in an awake model.

Ultrasound has been tested in awake rodents before, but most commonly with tFUS still delivered under anesthesia [39]. Wearable transducers for rodents have been developed, but generally have lower precision and parameter range, and only allow for EEG recordings [40–43]. To target an awake animal with tFUS precisely while taking high frequency recordings, head-fixation is required to keep the animal still. Head-fixation is typically done with mice due to training challenges [19, 44]. For tFUS however, the larger rat skull is preferred, providing enough space to insert an electrode and stimulate with ultrasound at the same time while avoiding creation of standing waves. Previous techniques for successful rat head-fixation [44, 45] are incompatible with tFUS delivery, covering too much of the skull with hard materials preventing ultrasound transduction. In addition, alignment must be achieved between the chronically implanted electrode within the headcap and the delivered ultrasound energy.

In the present study, we develop an awake head-fixed rat model in which intracranial recordings can be taken simultaneously with aligned ultrasound stimulation. Using this model, we tested a range of PRFs and DCs to determine their effectiveness on neuronal subtypes in the rat somatosensory cortex without the confound of anesthesia. By comparing these results to our own anesthetized ones as well as the numerous previous tests under anesthesia in the somatosensory cortex in literature [18, 34, 46], we demonstrate the cell-type specificity of tFUS and PRF and DC sensitivity in an awake model, showing that this effect is not due to secondary anesthesia interactions. Our results provide an enhanced understanding of the parameter space in awake rats, help us better understand the mechanism of tFUS induced neuronal activation, and demonstrate the potential of this model for use in future tFUS awake experimentation.

## 2. Methods

### 2.1. Awake head-fixed rat model

**Figure 1.**
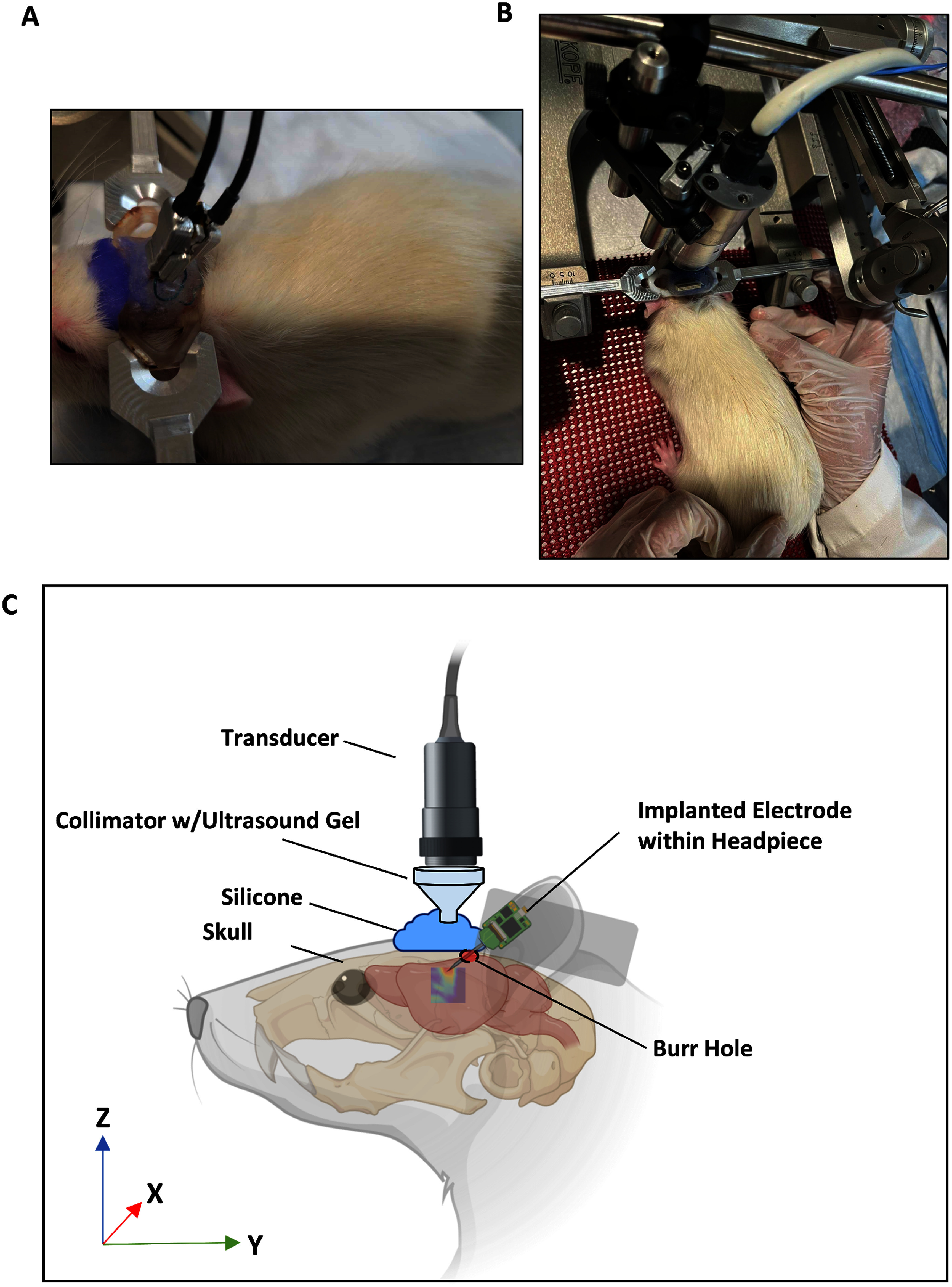
Awake head fixation set-up. (A) A picture showing the headpiece on a rat in head-fixation, with the ZIF-clip electrode and connector attached. (B) A picture showing the rat in head-fixation with the ultrasound transducer in position above. (C) A diagram showing the conformation of the transducer and ultrasound delivery relative to the rat.

15 adult male Wistar rats were used in this study, each weighing approximately 250 g. The animal study was conducted according to a protocol approved by the Institutional Animal Care and Use Committee (IACUC) of Carnegie Mellon University—approval: IPROTO202100000315, following the Public Health Service Policy on Humane Care and Use of Laboratory Animals. Surgery was performed to attach a headpiece and implant a 32-channel micro-electrode array. The headpiece used was developed from a previously published design [47] that has been successfully applied before [48]. Silicone was used to cover the electrode tip at the front while allowing ultrasound energy through, while screws and dental cement held the headpiece attached (figures 1(A) and (C)). After two weeks of recovery, animals were acclimated to head-fixation over a week before recordings began. By this point in training, rats sit entirely still for large stretches of time during the experiment and then go through brief periods of straining and moving, which would significantly increase the noise in the recording. Since these episodes were relatively isolated and very visibly obvious in data analysis, it was possible to manually ignore all neural activity during those episodes and analyze the rest. Keeping in mind the potential for movement artifacts however, we planned to have the rats sit for long sets of trials in order to minimize the effects of artifacts through averaging over a high number of trials. Male rats were selected in order to reduce the variance of S1 inhibitory neuron sensitivity which is known to vary with the estrus cycle of female rats [49]. Future studies using female rats may be beneficial given this variance. Details of headpiece design, surgery, and training protocols are given in the supplementary materials.

### 2.2. Ultrasound setup

Ultrasound was delivered from our 128-element random array ultrasound transducer H276 from (*f*_0_: 1.5 MHz, −3 dB focal size: 1.36 mm axially, 0.46 mm laterally), customized and manufactured by Sonic Concepts, Inc. (Bothell, WA, USA) (supplementary figure 2(A)). The transducer has a radius of acoustic aperture of 8.5 mm, and a 15 mm diameter of acoustic exit plane. Elements were arranged in a random distribution with 1 mm pitch (supplementary figure 2(B)). The transducer was driven with a Vantage 128 research ultrasound platform (Verasonics, Kirkland, WA, USA) using a DL-260 connector. A collimator was designed to attach to the transducer, and was 3D printed with VeroClear material. The collimator was filled with ultrasound gel (Aquasonic Ultrasound gel, Parker Laboratories, Inc., Fairfield, NG, USA) and attached to the transducer before experiments. The collimator and transducer were aligned with the targeted brain region, and the Verasonics system was used to steer the ultrasound beam’s depth through the collimator and into the brain at S1 (coordinates *X* = 0, *Y* = 0, *Z* = 35, relative to the center of the transducer’s spherical cap).

**Figure 2.**
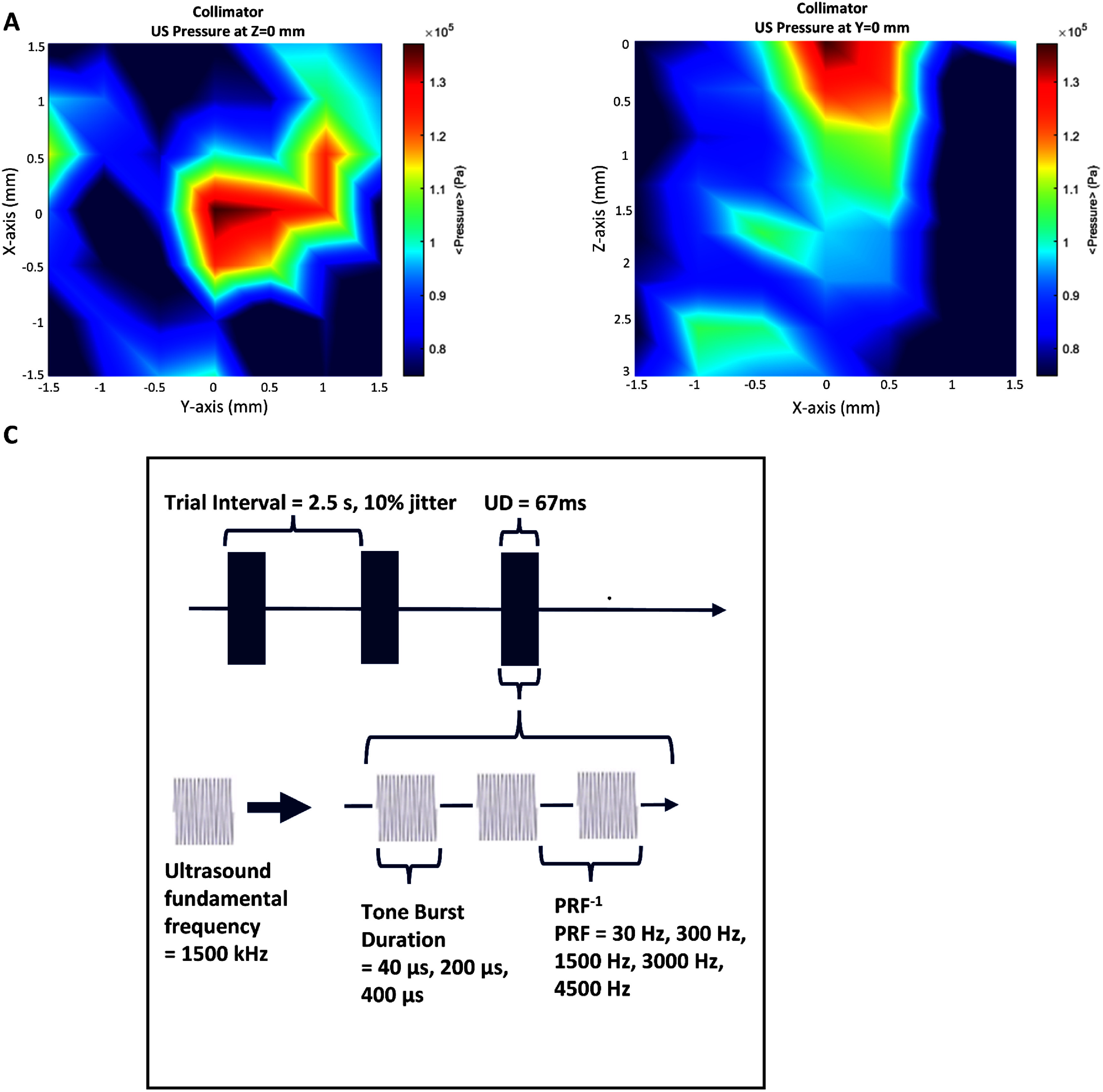
Ultrasound field characterization and delivery parameters. (A) *Ex-vivo* hydrophone peak-to-peak pressure amplitude *z*-axis scan at the expected depth of the targeted brain region. (B) *Ex-vivo* hydrophone peak-to-peak pressure amplitude *y*-axis scan at the expected position of the targeted brain region. The measured *I*_SPTA_ here using 1500 Hz PRF and 67 ms PD was 23.7071 mW cm^−2^, and the *I*_SPPA_ is 79.0238 mW cm^−2^. Beam plots for (A) and (B) are shown in supplementary figure 4. (C) Diagram of the ultrasound delivery waveform parameters used in stimulation trials. The fundamental frequency of 1500 kHz on the bottom left is pulsed, at a variety of frequencies called the ‘pulse repetition frequency’ (PRF) and with varying tone burst durations (TBD). A train of these pulses is delivered for 67 ms, the ultrasound duration, in each trial. Trials are initiated every 2.5 s.

*Ex-vivo* scanning was performed using a customized needle hydrophone-based 3D ultrasound pressure mapping system (HNR500, Onda Corporation, Sunnyvale, CA, USA) with an extracted rat skull sample with a headpiece attached, which allowed us to measure the delivered pressure in our setup. We determined that an input voltage of 15 V resulted in a measured peak-to-peak ultrasound pressure of 114 kPa in the targeted region after penetrating the skull and silicone layer while using parameters of 1500 Hz PRF, a 67 ms pulse duration (PD), and a 200 *µ*s tone-burst duration (TBD) (figures 2(A) and (B)). The measured *I*_SPTA_ result was 23.7071 mW cm^−2^, and the *I*_SPPA_ is 79.0238 mW cm^−2^. Our scan in figure 2(A) shows a deflection in the *+Y* direction, likely resulting from a slight deformity in the skull and silicone above it or from miniature air bubbles within the collimator, illustrating how much these slight physical variations can affect the ultrasound beam. An *ex vivo* scan taken with no skull/silicone layer and no collimator is presented in supplementary figure 2 illustrates the un-deformed beam. Details of the scanning process are provided in the supplementary materials.

### 2.3. Experimental design

We tested 10 rats implanted with chronic electrodes and trained for head fixation, taking recordings of stimulation at the 5 PRF levels previously used by our group, 30 Hz, 300 H, 1500 Hz, 3000 Hz, and 4500 Hz, while using a TBD of 200 *µ*s, in our awake model and then in the same 10 rats under anesthesia in order to confirm that the results under anesthesia within our model were similar to previous acute studies [18, 34] (figure 2(C)). Based on our *ex vivo* measurement of peak-to-peak pressure, we calculated that the PRF conditions must have an *I*_SPTA_ of 0.6 mW cm^−2^, 6.03 mW cm^−2^, 30.13 mW cm^−2^, 60.26 mW cm^−2^, and 90.39 mW cm^−2^ respectively by calculating the pulse intensity integral ${\text{PII}} = {\mathop \smallint \nolimits }{}\frac{{{p^2}\left( t \right)}}{{{Z_0}}}{\text{d}}t{ }$ and then multiplying by the different values for PRF. We then held PRF constant at 1500 Hz and tested TBD of 40 *µ*s, 200 *µ*s and 400 *µ*s, resulting in DCs of 6%, 30% and 60%, respectively. Each parameter combination was tested by delivering 505 trials of ultrasound stimulation, with one trial every 2.5 s. The recording software was used to send a trigger to the transducer at this time interval with a jitter of 10% in order to minimize any brain adaptations to tFUS stimulation. Analysis did not include the time window with this variation, and the precise trigger times of ultrasound were stored alongside the continuous data for future analysis. The ultrasound duration of each trial was 67 ms, and all trials used an *f*_0_ of 1.5 MHz. 10% jitter was used in the inter-stimulus interval timing to minimize any brain adaptation to tFUS stimulation. Recordings for PRF tests were completed first, while rats were approximately 7 months old, 1 month after surgery. Anesthetized recordings were completed when rats were 9 months old, and recordings for DC tests at 10 months. Recordings for a single rat for each set of parameters were completed on the same day, with either 2 or 3 recordings completed during each head-fixation session. We then implanted 5 more rats with chronic electrodes for head fixation, and at 7 months of age completed both awake and anesthetized recordings using 30 Hz and 1500 Hz PRF stimulation with 200 *µ*s TBD, as well as a sham condition at 1500 Hz PRF with the ultrasound beam directed off target towards the front of the skull. This allowed us to confirm that any difference between the conditions we observed was not due to the time gap between our awake and anesthetized recordings. 300 Hz and 1500 Hz were selected because we expected to observe responses to tFUS in both as well as a clear difference in response level. The sham condition allowed us to confirm that the activation we observed was not due to any auditory confound from off-target activation or skull conduction from the transducer.

### 2.4. Electrophysiology

**Figure 3.**
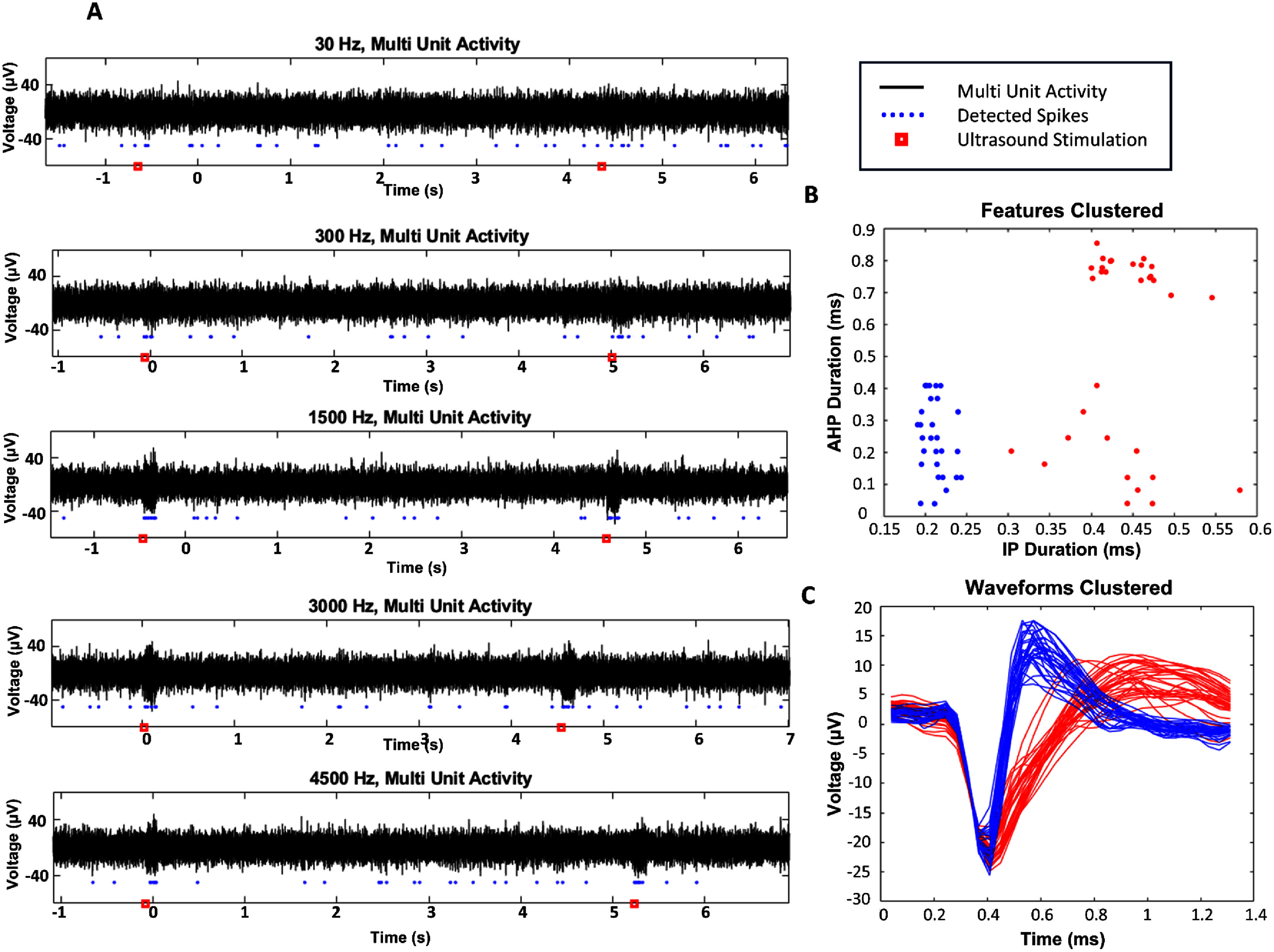
Multi-unit activity (MUA) response to tFUS and cell-type sorting. (A) Examples of recorded MUA from our awake head-fixed setup with tFUS delivered at five different PRFs (30 Hz, 300 Hz, 1500 Hz, 3000 Hz, 4500 Hz) in trials. The blue dots underneath the signal in black are the sorted spikes from each section of signal. (B) *K*-means clustering of detected neurons from one animal’s recordings, showing 28 fast spiking units (FSU) and 32 regular spiking units (RSU), for 60 total. On the *x*-axis is plotted the initial phase (IP) of the action potential’s duration, and on the *y*-axis is the after-hyperpolarization (AHP) phase of the action potential’s duration. Blue dots were deemed to be FSU, and red dots were considered to be RSU. (C) Plotted average action potential waveforms for each of the clustered neurons shown in (B). Blue waveforms are FSU, while red waveforms are RSU.

Recordings were taken using the TDT Neurophysiology Workstation (24 kHz sampling frequency, Tucker-David Technologies, Alachua, FL, USA). Neural trace was bandpass filtered between 300 Hz and 5 kHz for spike analysis. Characteristic raw neural trace responses to tFUS alongside detected spikes, and the signal with noise filtered out using are shown in figure 3(A). All experimental data has been made public in a repository at the DANDI Archive [50]. Spike sorting was performed using the PCA-based spike classification software Offline Sorter (Plexon, Dallas, TX, USA). Manual spike sorting involved first removing high amplitude noise as well as cross-channel artifacts using automatic tools in the software. Automatic clustering was then performed using the software’s expectation-maximization algorithm. Any clusters determined to be noise due to their improper waveform shape were removed. Clusters with peak positive amplitude no higher than baseline were also removed, as well as any clusters with spikes not present through the entire recording or with ISI histograms showing periodicity or lack of a refractory period, indicating multi-unit activity.

After sorting, peristimulus time histograms (PSTHs) and raster plots were computed for each neuron in MATLAB R2019a (The MathWorks, Inc., Natick, MA, USA) using the FieldTrip toolbox [51] and custom code. PSTHs were computed from 0.5 s prior to ultrasound stimulation, until 1.5 s afterwards, and then divided by the number of trials (i.e. 500 after 5 are cut from the beginning of the recording), resulting in a histogram of average spikes per time bin for each neuron. Baseline was computed from the 0.5 s before tFUS onset, and the response to tFUS was characterized as the normalized spike rate as a percent of baseline within the specified time span. All neurons were included in this analysis without considering if a given neuron showed a response significantly different from baseline activity. Any PSTHs with visibly fluctuating baseline periods were excluded from analysis and considered to be contaminated by artifacts.

We first characterized the previously studied time-locked response, defined as the response during the 67 ms ultrasound stimulation, and then delayed responses, defined as any response after the ultrasound duration. To analyze latency of delayed responses, neurons with significant response were first selected, characterized as those with at least three consecutive time bins in their PSTH with value greater or equal to one SEM above baseline. Latency was then characterized as the time bin of the first of the three or more time bins.

### 2.5. Cell subtype sorting

Neurons were sorted into fast spiking units (FSU) and regular spiking units (RSU) using custom code in MATLAB, which are putative inhibitory interneurons and excitatory pyramidal neurons respectively, using the features of their action potential waveforms [52–59]. The specific features used were the duration from onset to first crossing of baseline (initial phase), and the duration from first crossing of baseline to recrossing of baseline (after-hyperpolarization phase). *K*-means clustering was used to sort the neurons based on these features (figures 3(B) and (C)). In cases where neuronal features were not clearly in one cluster, spiking rate was used to further aid in sorting, and generally these were found to be RSU.

### 2.6. Statistical analysis

Responses of the different neuronal sub-groups within each condition were compared to test whether PRF and DC are significant factors, and to find which parameters elicit highest responses. Neuronal response data were tested for normality using the Shapiro–Wilk test, and were found not to have a normal distribution. Given this, the Kruskal–Wallis H test was used for group analysis of significance, and characterization was performed with post hoc Wilcoxon tests. The Bonferroni correction for multiple comparisons was used when examining *p*-values for significance. Results of statistical tests are given in the Results section. The N used for these tests is the total number of neurons in each group, across all animals (ten animals for the initial recordings, and five for the follow-up recordings), and is given in each figure below the relevant group. Parameters were recorded in a random order in each animal.

## 3. Results

### 3.1. Differences in response latencies between awake and anesthetized models

**Figure 4.**
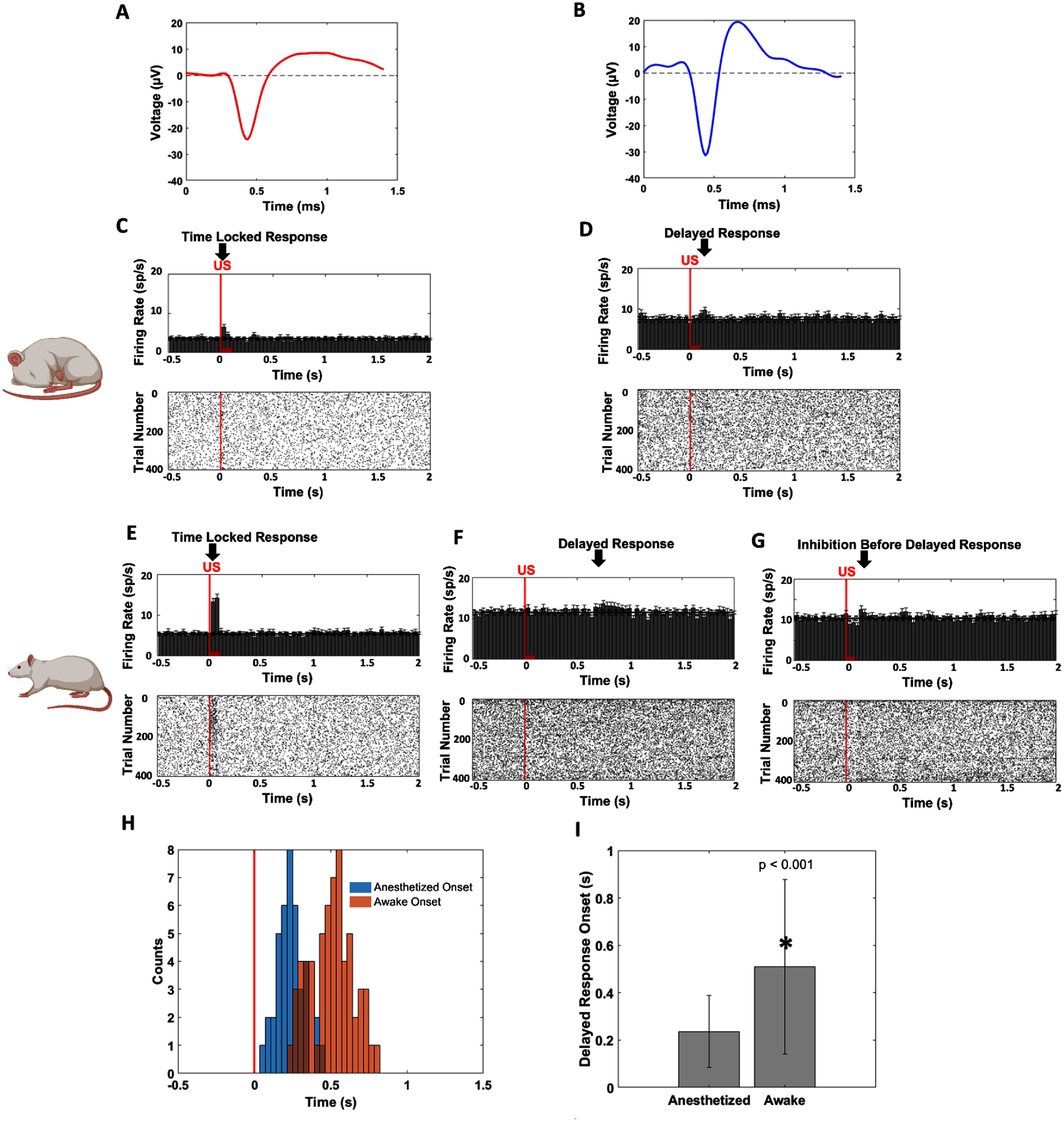
Varying latencies in cell-type specific neuronal spiking responses to tFUS in awake and anesthetized models. (A) A characteristic waveform of an RSU from acquired recordings. (B) A characteristic waveform of a fast spiking unit from acquired recordings. (C)–(G) Representative peristimulus time histograms (PSTHs, bin size: 35.7 ms, *n* = 505 trials for each time bin) and raster plots of responses to trials of 1500 Hz PRF 30% DC tFUS from (C) an RSU in the anesthetized model, (D) an FSU in the anesthetized model, (E) an RSU in the awake model, and (F) and (G) two different FSU in the awake model. The vertical red line shows the tFUS onset. (H) Plotted histograms of FSU delayed response onset latencies at 1500 Hz PRF in the anesthetized model (blue) and the awake model (orange). The horizontal red line shows the tFUS onset. (I) A bar plot showing the difference in average delayed response onset latency between the awake and anesthetized model. Significance was characterized with a Wilcoxon rank sum test, and the groups were found to be significantly different with *p* < 0.0001.

We first examined the differences in neuronal responses to tFUS between awake and anesthetized recordings. We observed time-locked responses from RSU in both awake and anesthetized recordings, with an observable increase in firing rate during tFUS stimulation (figures 4(C) and (E)). From FSU we observed delayed responses to tFUS after the 67 ms of stimulation in both awake and anesthetized recordings, but response onset latencies of this response varied between the models (figures 4(D), (F) and (G)). In the anesthetized model, these delayed responses had an average onset latency of 0.24 s ± 0.08 s, while in the awake model they had an average onset latency of 0.51 s ± 0.14 s (figure 4(I)). The awake model had a wider variance of onset latencies, and appears to have two peaks (figure 4(H)). FSUs in awake rats generally had a wider variety of responses. Figure 4(F) shows the higher latency response that was most commonly observed, while figure 4(G) shows an earlier latency response more similar to that observed in the anesthetized model, and time locked inhibition. Comparing the distributions using the Wilcoxon rank-sum test, we found that delayed response onset times varied significantly between awake and anesthetized models (*p* < 0.001).

### 3.2. Awake vs anesthetized model sensitivity to PRF

**Figure 5.**
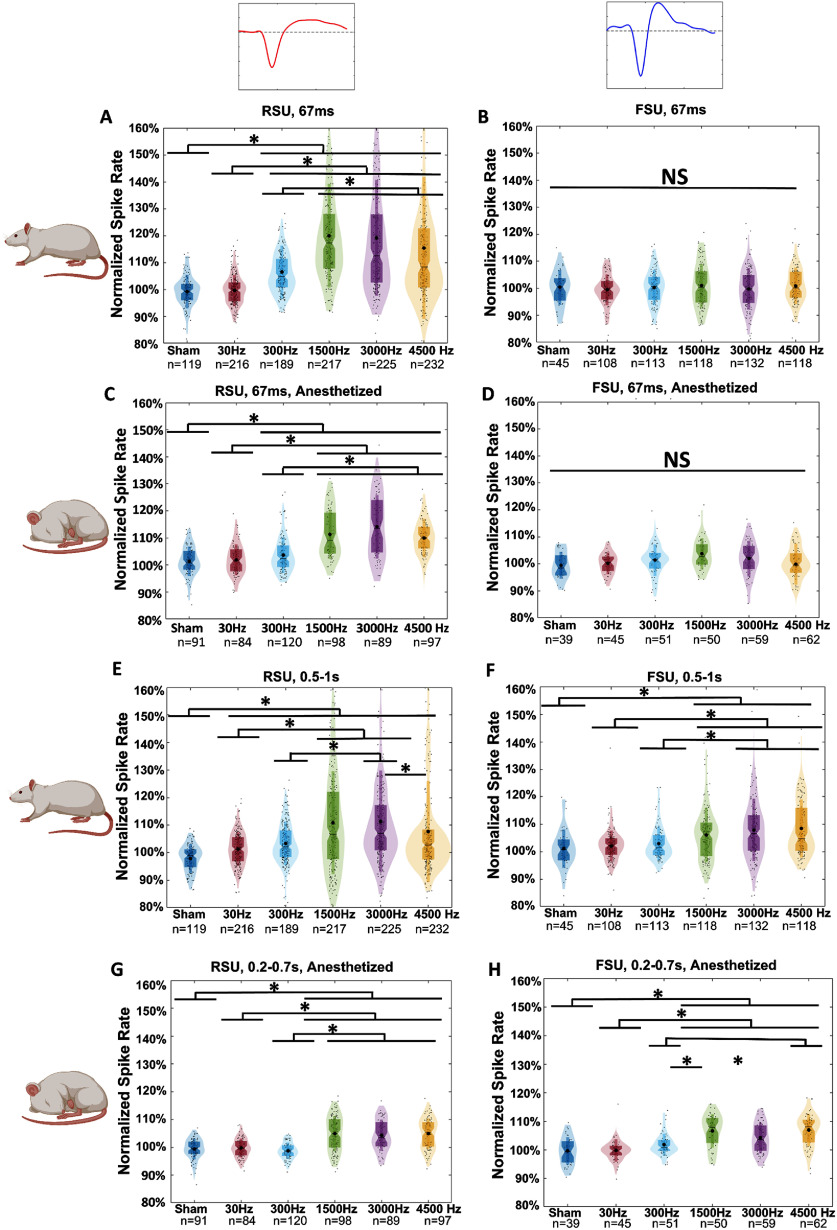
Group analysis of cell-type specific responses to tFUS with varying PRF in awake and anesthetized models. (A)–(H) all show violin plots of the average normalized spike rate induced by tFUS within each parameter over 505 trials in the specified time range compared to baseline, calculated from the 0.5 s before stimulation. Each small black dot represents a neuron from a recording under the specified parameters. The black asterisks show the mean of each group. The darker color figures are traditional box plots with the ends of the boxes marking the 1st and 3rd quartiles and the middle line marking the median. Notches mark the 95% confidence intervals on the median. The narrow rectangle edges mark one standard deviation to each side of the mean of the group. The lighter colored figures are the probability distribution of the data, with the ends of the figure marking the 1st and 99th percentile. Statistics are performed using the Kruskal–Wallis two-sided one-way ANOVA on ranks, with *post -hoc* Wilcoxon tests with the Bonferroni correction for multiple comparisons used afterwards. (A) Time locked responses from RSU in the awake condition. Significantly different responses to different PRFs are observed. (B) Time locked responses from FSU in the awake condition. No significant differences are observed. (C) Time locked responses from RSU in the anesthetized condition. Significantly different responses to different PRFs are observed. (D) Time locked responses from FSU in the anesthetized conditions. No significant differences are observed. (E) Delayed responses (0.5–1 s) from RSU in the awake condition. Significantly different responses to different PRFs are observed. (F) Delayed responses (0.5–1 s) from FSU in the awake condition. Significantly different responses to different PRFs are observed. (G) Delayed responses (0.2–0.7 s) from RSU in the anesthetized condition. Significantly different responses to different PRFs are observed. (H) Delayed responses (0.2–0.7 s) from FSU in the anesthetized conditions. Significantly different responses to different PRFs are observed.

We tested PRFs of 30 Hz, 300 Hz, 1500 Hz, 3000 Hz, and 4500 Hz while holding the TBD at 200 *µ*s in order to determine whether cell type selective responses and PRF sensitivity are maintained in an awake model. Examining the time-locked response from RSU in the awake model using Kruskal–Wallis ANOVA, we find that PRF is a significant factor (*p* < 0.001). Post hoc tests show that 30 Hz and 300 Hz PRF induced responses lower than all higher PRFs (*p* < 0.001). The sham condition was significantly different than all but the 30 Hz condition (*p* < 0.001). 1500 Hz, 3000 Hz, and 4500 Hz showed no significant differences (figure 5(A)). The anesthetized model had similar statistical results, with the only difference being that no significant difference was observed between 30 Hz and 300 Hz PRF levels (figure 5(C)). Two-way ANOVA additionally finds that anesthesia is a significant factor in the time-locked response from RSU (*p* < 0.01). FSU in both the awake and the anesthetized model show no significant differences due to PRF levels (figures 5(B) and (D)).

Next, we considered the delayed responses. PRF was found to be a significant factor for RSU and FSU in both awake and anesthetized settings (figures 5(E)–(H)). RSUs in the awake rats had highest responses to 1500 Hz and 3000 Hz (*p* < 0.001), with a decrease at both higher and lower frequencies, and all conditions found to be significantly higher than sham (*p* < 0.001) (figure 5(E)). FSUs in the awake model show the strongest responses to 4500 Hz PRF, with significant differences between 30 Hz and the highest three PRFs, and 300 Hz and the highest two PRFs. (*p* < 0.001, figure 5(F)). 30 Hz and 300 Hz were not found to be significantly higher than sham. Both RSUs and FSUs in the anesthetized rats had similar sensitivity as their time-locked responses, broadly preferring higher PRFs (figures 5(G) and (H)). Using two-way ANOVA, we found that anesthesia is a significant factor in the delayed response for both RSU (*p*< 0.001) and FSU (*p* < 0.01).

**Figure 6.**
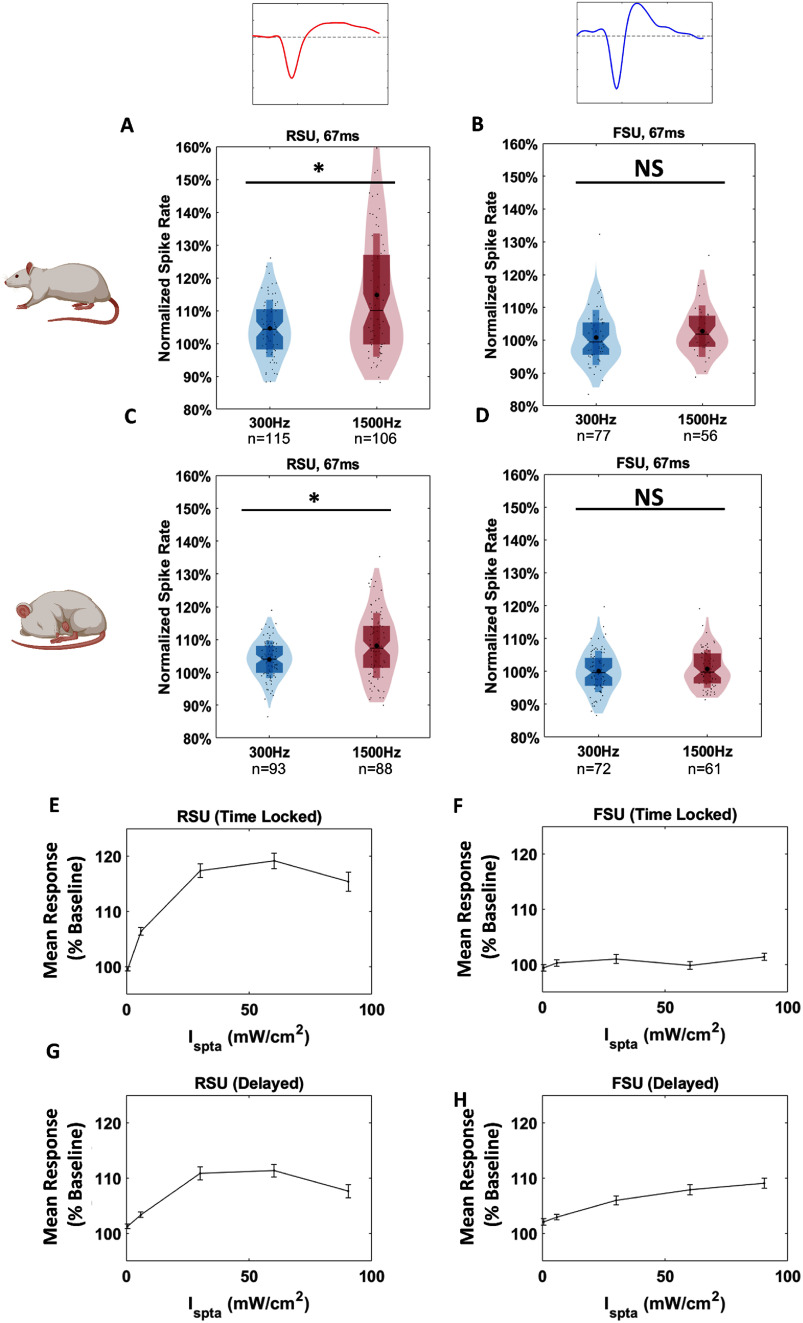
Group analysis of time-adjacent recordings and responses to varying *I*_SPTA_ (A)–(D) show violin plots for each condition of the average normalized spike rate induced by tFUS over 505 trials from 0–67 ms compared to baseline, calculated from the 0.5 s before stimulation. Statistics are performed using the Kruskal–Wallis two-sided one-way ANOVA on ranks, with *post -hoc* Wilcoxon tests with the Bonferroni correction for multiple comparisons used afterwards. These plots repeat figures 5(A), (B), (E) and (F) with only 2 conditions in 5 new animals, taking both anesthetized and awake recordings in the same week to ensure similar signal quality (E) (F) (G) and (H) show plots of mean response taken from figure 5 plotted against delivered *I*_SPTA_ in RSU and FSU, with standard error of the mean shown. (E) and (F) show the time locked response within 67 ms for RSU and FSU respectively. (G) and (H) show the delayed response, from 0.5–1 s for RSU and FSU, respectively.

To confirm that the observed differences between awake an anesthetized conditions was not due to the time gap, we next analyzed data from an additional batch of five rats in which awake and anesthetized conditions were recorded in the same week, one month post-surgery. In analysis of the time locked responses, we observed significant differences in response to 300 Hz PRF and 1500 Hz PRF stimulation in both awake and anesthetized conditions (*p* < 0.05), but with a reduced response under anesthesia (figures 6(A)–(D)).

We next plotted the mean responses of RSU and FSU against *I*_SPTA_, converted from our parameters, and plotted the mean responses from the awake animals against it (figures 6(E)–(H)). We observe that RSU’s in both their time-locked and delayed responses initially increase till around 60 mW cm^−2^, after which the response declines. FSU’s show no time-locked response, and their delayed response increases almost linearly with *I*_SPTA_.

Overall, 1500 Hz, 3000 Hz, and 4500 Hz produce significantly higher responses, with no significant differences among them. Although it is not statistically significant, we found that 1500 Hz produces the highest mean time-locked response (figure 5(A)).

### 3.3. Awake model sensitivity to DC

**Figure 7.**
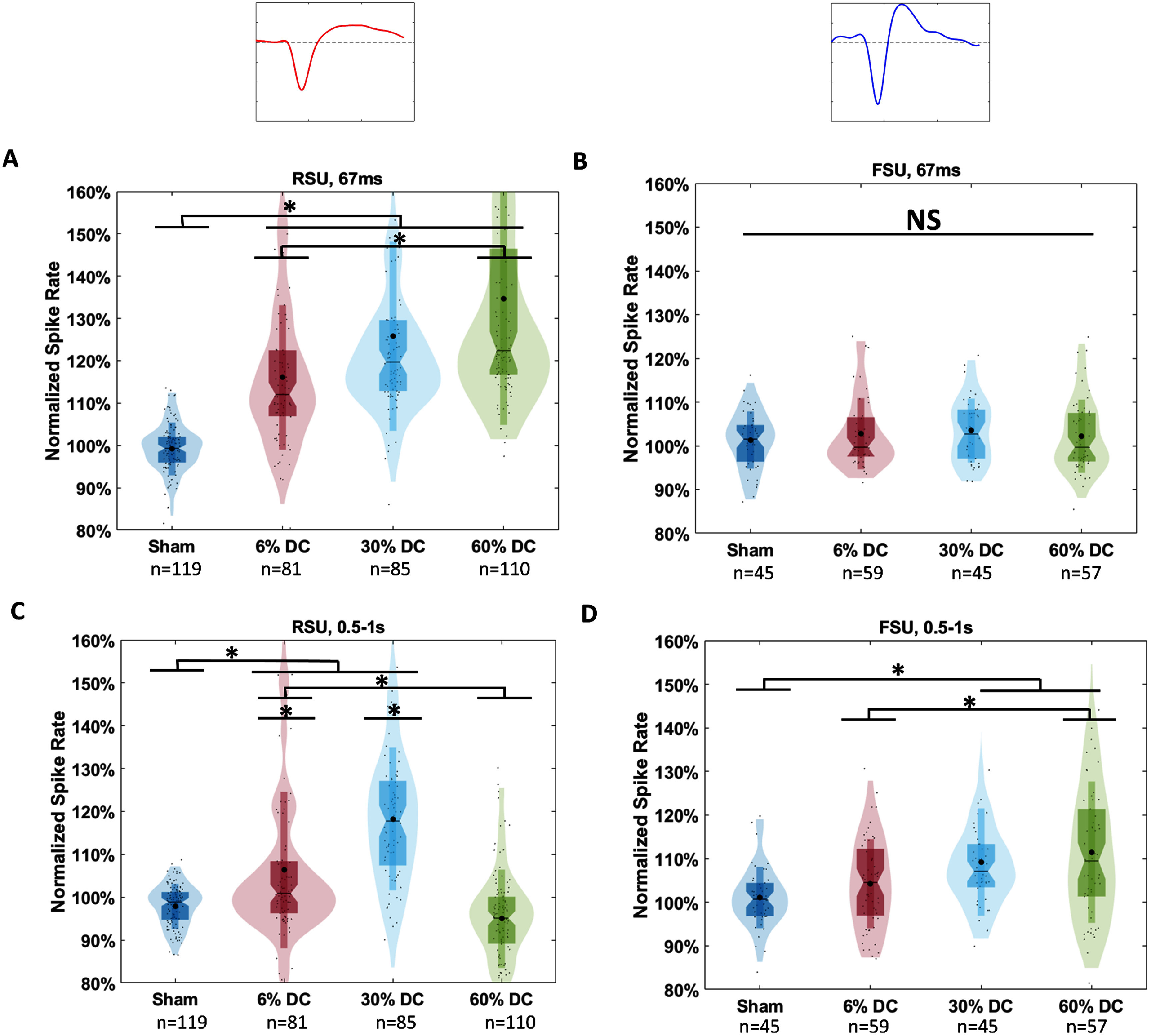
Cell-type specific responses to varying duty cycle of tFUS in the awake model. (A)–(D) Show violin plots for each condition of the average normalized spike rate induced by tFUS over 505 trials in the specified time range compared to baseline, calculated from the 0.5 s before stimulation. Statistics are performed using the Kruskal–Wallis two-sided one-way ANOVA on ranks, with *post -hoc* Wilcoxon tests with the Bonferroni correction for multiple comparisons used afterwards. (A) Time locked responses to DC among RSU. 6% DC results in a significantly lower response than 30% or 60%. (B) Time locked responses to DC among FSU. No significant differences are observed (C) Delayed responses (0.5–1 s) to DC among RSU. Significantly different responses are observed between all DCs. (D) Delayed responses (0.5–1 s) to DC among FSU. 6% and 60% DC show significantly different levels.

Following the previous results, we next tested DC sensitivity by changing the TBD while holding PRF constant at 1500 Hz. Time-locked responses from RSUs showed a significantly lower response to 6% DC than those to 60%, with all conditions significantly higher than sham (*p* < 0.001, figure 7(A)). Delayed responses however preferred 30% DC over 6% and 60% (*p*< 0.001, figure 7(C)), and 60% DC showed no significant difference from sham. Delayed responses from FSUs showed a significantly higher response to 60% DC over 6% (*p* < 0.001, figure 7(D)) with 6% DC found to be not significantly different from sham, and no time-locked response was observed as in the PRF study (figure 7(B)). Based on mean values, 60% DC appears to produce the strongest time-locked response, although it is only statistically significantly higher than the 6% condition.

## 4. Discussion

In this study, we have developed an awake head-fixed rat model to simultaneously deliver ultrasound stimulation and take multi-electrode intracranial electrophysiological recordings. Using this novel animal model, we have investigated a variety of ultrasound parameters to test our hypothesis that tFUS would induce cell-type specific neuronal responses to both PRF and DC variation. Our study of the ultrasound parameter space without the confound of anesthesia provides a better understanding of the mechanisms behind tFUS neuromodulation in different cell-types in an awake animal model.

The developed model allowed us to take multi-electrode intracranial electrophysiological recordings with high spatial and temporal specificity while delivering ultrasound stimulation. This allowed us to record spiking activities of awake rats responding to ultrasound stimulation for the first time to the best of our knowledge. This setup does not require any specialized transducer, 3D printed headpieces, and utilizes a regular stereotaxic setup. Weaknesses of this model as currently developed are noise management and targeting precision. To ensure minimal noise, the recording room needs to be electrically isolated, and grounding during surgery for each animal needs to be performed carefully. Targeting relies on careful alignment of the electrode during surgery and of the transducer in each recording session, which is a potential source of variation. By contrast, common acute electrode insertion recordings performed under anesthesia tend to have less noise and may have a larger number of neurons in each recording, as well as much better targeting precision, but do not allow for any behavioral testing in addition to the anesthesia confound. Another model growing in popularity is the use of two-photon calcium imaging as it allows the analysis of confirmed single units of different cell types, however this model does not allow for testing of deeper regions of the brain. Despite this, we consider this model a useful new tool for future testing of neuronal responses to tFUS neuromodulation in an awake setting for rat models, making future behavioral testing simpler as well.

Using this model, we recorded awake neuronal responses to tFUS in the primary somatosensory cortex (S1), testing different PRF levels and DCs. The results provide *in vivo* evidence that RSU responds in a time-locked manner that is sensitive to increases in PRF in awake animals. Anesthetized recordings also show this effect, aligning with previous studies [18]. FSU shows no significant time-locked response to PRF changes in either awake or anesthetized recordings. This demonstrates that the cell-type differentiated effect is not due to anesthesia effects. Delayed responses to tFUS in both cell-types were observed to be sensitive to PRF level as well. These responses first increased with PRF before then declining at a higher PRF, 4500 Hz. Similarly, RSU time-locked responses preferred higher DCs, while their delayed responses were strongest in the middle range. By contrast, FSU delayed responses were strongest at 60% DC. This demonstrates the cell type specificity of DC variation. Observation of PRF and DC sensitivity in both the time-locked and delayed response shows that they are likely caused by the same mechanism. The difference in preferred parameters between the time-locked and delayed responses however indicates that there may be other factors affecting the magnitude of delayed responses.

Sensitivity to combinations of both PRF and DC suggests that the delivered *I*_SPTA_ and thus dynamic acoustic radiation force (ARF) may be a significant mechanism of ultrasound neuromodulation at play. ARF as it applies to tFUS is the force resulting from attenuation of momentum of the ultrasound wave into the brain tissue. Mechanical force such as this stretches cell membranes and modulates ion channel conformations as well as their fluid environments, altering membrane conductance and thus spiking probabilities. The magnitude of the ARF is determined by the temporal average intensity and can be calculated at the spatial peak with the *I*_SPTA_, which further depends on PRF and DC/TBD, which in combination control the delivered energy dose [18]. Calculations of these values from our parameters can be found in supplementary table 1.

In our plots of RSU and FSU responses to *I*_SPTA_, as proportional to PRF, we observe that RSU responses initially increase before saturating and beginning to decrease. FSUs, however, have no time locked response, but a more linear delayed response. Recent research using calcium imaging has suggested that while PV+ neurons (FSU, inhibitory) show no response at low intensities before, Thy1+ (RSU, excitatory) and SST+ (RSU, inhibitory) show increases immediately, SST+ having a stronger response at lower intensities before declining at higher intensities [60]. Since RSUs may include some of these inhibitory neurons alongside the majority excitatory neurons, the dynamics of both may affect the responses observed, and be the source of the decline at higher intensities.

As previously discussed, neuronal subtypes express different ion channel distributions, shapes, and dendritic arbor alignments within the cortex. We hypothesized that these differences were the cause of the cell-type specific sensitivity to PRF changes, in relation to ARF. In this study, we confirm that this cell type specificity does not come from cell-type specific anesthesia effects, providing further evidence that ARF may be responsible for this difference.

Although these results demonstrate that anesthesia is not a confound to cell-type specificity, modulation of responses by anesthesia was observed. Anesthesia was observed to decrease the average magnitude of RSU responses. FSU however exhibited more varied response dynamics overall in their delayed response, in addition to an average decrease in response magnitude. Although previous analysis has focused on time-locked responses to tFUS, delayed responses have been observed before. Tufail *et al* observed a smaller delayed response at approximately 250 ms post-stimulation in anesthetized mice [10]. Murphy *et al* used calcium imaging to investigate cell-type specific responses to tFUS and found that PV+ inhibitory interneurons exhibited a slow increase in activity 1–3 s after stimulation [36]. Our group previously observed no delayed responses from FSU while using a ketamine/xylazine cocktail as anesthesia, which had significantly modulated responses relative to later isoflurane recordings [18]. Fewer FSUs were recorded under ketamine/xylazine compared to isoflurane, indicating that it may have a particularly dampening effect on FSU. Our group’s more recent research has indeed observed delayed responses in FSU under isoflurane anesthesia, although we still did not observe the later delayed responses seen in awake recordings [61].

The mechanism behind the delayed responses is not fully clear. Murphy *et al* hypothesized that PV+ cells have a different mechanism causing them to respond to tFUS in a delayed manner, or that it may be a polysynaptic effect. Our group has hypothesized that these delayed responses are most likely due to cortico-thalamo-cortical (CTC) circuit activation and feedback [61]. Under this hypothesis, tFUS activates excitatory neurons in S1, which then activate neurons in the thalamus. These further activate both excitatory and inhibitory neurons in S1, inducing delayed responses. It has been demonstrated that CTC connectivity is significantly reduced by anesthesia in rats, which may explain the difference in the awake model [19, 29]. Previous studies of electrical stimulation in the cortex have shown delayed (or ‘rebound’) responses due to CTC activation, which were muted while under anesthesia [62]. Although the mechanism behind ultrasound stimulation activation is different than electrical stimulation activation, this same dynamic may be at play.

Another potential explanation is that higher latency responses observed from FSU in the awake model may be due to a different mechanism of ultrasound activation which is blocked under anesthesia. This mechanism may be more in line with the slow PV+ activation observed by Murphy *et al*, also in awake animals [36]. Under the theory of an ARF mechanism, the ion channel distribution and geometrical shape of RSU may result in quick elicitation of activity, while the ion channel distribution and shape of FSU may result in a slower elicitation that can be blocked by anesthesia effects on those ion channels.

One limitation in our study is the number of animals used. Ultrasound is known to have particularly variable effects. There is high inter-subject variation, and trial to trial, stimulation is unlikely to have the same response each time. Orientation of different neurons to the ultrasound wave may affect how they are stimulated. Numbers in this study were limited due to the challenge of training and maintaining AHF model animals over months, but future studies may use more animals to clarify differences between parameter levels. Another limitation was the range of parameters tested. This study kept the fundamental frequency, intensity level, and ultrasound duration constant, and did not test variation of PRF and TBD while holding DC constant. This was done for comparison to our group’s previous anesthetized study, and because we are particularly interested in time-locked excitation’s potential to induce plasticity within a certain delivery paradigm [34].

We also limited these recordings to one region of the brain, the somatosensory cortex. This region was best suited for our study so that we could compare to previous studies. In general, the somatosensory cortex is a desirable target since it is a well understood region as well as shallow within the brain, allowing for efficient testing and has a variety of excitatory and inhibitory neuron types [63]. Additionally, tests here allow us to move towards future evaluation of behavioral responses to physical stimuli and pain perception, as well as tests of therapies for chronic pain [39, 64]. Although we expect the general trends of responses to be consistent across the brain, and in particular that increased PRF leads to increased response, optimal parameters may vary between brain regions. There is plentiful evidence that effects vary between cell types, which vary between regions of the brain. Effects are known to vary between cell types, the distributions of which fluctuate across brain regions. There is also evidence that effects can vary even between layers of cortex [34]. Future research will have to include other regions of the brain as well as exploring capabilities for simultaneously targeting multiple regions.

One potential confound in ultrasound neuromodulation studies is indirect auditory activation [65, 66]. In our previous anesthetized study with similar setup [18] we tested several sham conditions that would induce similar noise or skull vibration, and did not observe any significant response. In this study we additionally performed a sham condition delivering ultrasound off target to the front of the skull, and did not observe any significant response. Based on this, we consider this not to be a confound to our results. Another concern is whether the electrode itself interferes with the delivery of ultrasound, particularly whether any vibration is caused. Positioning the ultrasound opposite to the electrode’s insertion as we do should ensure that the recorded brain is receiving unblocked stimulation, and we do not expect electrode vibration effects to be a confound at our delivered pressure level of 114 kPa, under the threshold at which this might be a concern [67].

## 5. Conclusion

This study confirms the cell-type specific and parameter-dependent qualities of ultrasound in an awake rodent model. We observed that RSUs responded to tFUS in a time-locked manner, while FSUs responded at a delayed latency, that was modulated by anesthesia. Both these responses showed sensitivity to PRF and DC. These results provide further evidence that ARF plays a key role in the tFUS neuromodulation. Although our findings indicate that anesthesia was not a major confound to the observations of these characterizations of tFUS-neuron interactions, we also observe that anesthesia significantly modulates the response to tFUS, and that FSU delayed responses show modulated dynamics under anesthesia. Future investigation of tFUS effects on circuit dynamics or different cell types should consider this confound. Our developed AHF rat model for tFUS will allow for intracranial electrophysiological investigation into tFUS without anesthesia in the future.

## Data Availability

All data that support the findings of this study are included within the article (and any supplementary files).
